# Ambient Artificial Intelligence Scribes in Pediatric Hematology—Oncology: Early Implementation of DAX Copilot

**DOI:** 10.1055/a-2873-3894

**Published:** 2026-05-22

**Authors:** Molly Talman, Lamia Alam, Dara Mize, Yaa Kumah-Crystal, Carolynn Nall, Allison B. McCoy, Adam Wright, Laura Zahn, Jennifer Andrews

**Affiliations:** 1 12328 Department of Pediatrics, Vanderbilt University Medical Center, Nashville, Tennessee, United States; 2 12328 Department of Biomedical Informatics, Vanderbilt University Medical Center, Vanderbilt University, Nashville, Tennessee, United States; 3 12328 Department of Medicine, Vanderbilt University Medical Center, Nashville, Tennessee, United States; 4 12328 Department of Pathology, Microbiology and Immunology, Vanderbilt University Medical Center, Nashville, Tennessee, United States

**Keywords:** artificial intelligence, clinical documentation and communications, electronic health records and systems, diffusion of innovation, communications

## Abstract

**Background:**

Ambient artificial intelligence scribes are increasingly integrated into electronic health records to reduce documentation burden, but limited data describe their performance in high-complexity pediatric subspecialties.

**Objectives:**

This study aimed to describe the early implementation of Dragon Ambient Experience (DAX) Copilot in a pediatric hematology–oncology division and evaluate patterns of use, documentation time, and workflow effect among providers who adopted the tool.

**Methods:**

We reviewed outpatient encounters from January to July 2025 and identified those using DAX. Analyses focused on providers who used DAX more than twice, given low overall adoption. Documentation time (minutes spent in active editing sessions) and note-closure timeliness were compared between DAX and non-DAX encounters. Implementation processes, barriers, and accuracy concerns were qualitatively summarized.

**Results:**

Of 11,544 outpatient encounters, 427 (3.7%) involved DAX; 10 of 29 providers used DAX at least once, while 6 (20.7%) used it more than twice. Among repeat users, for shorter encounters (≤40 minutes), median documentation time was lower for DAX versus non-DAX (11 [interquartile range, (IQR): 13] vs. 24 [IQR: 30] minutes), corresponding to an approximately 30% lower documentation time (95% confidence interval [CI], 19.9–38.4%; *p* < 0.001). For longer encounters (>40 minutes), documentation time did not differ significantly. Although unadjusted analyses showed lower rates of same-day and 7-day note closure with DAX, these differences were not significant after adjustment in mixed-effects logistic regression models. Providers reported improved engagement with families and narrative drafting; limitations were related to workflow alignment rather than transcription accuracy.

**Conclusion:**

For repeat users, DAX reduced active documentation time in shorter visits but showed limited benefit for longer encounters and did not improve the timeliness of note closure. Adoption remained modest, underscoring the importance of workflow fit and usability considerations in AI-scribe deployment.

## Background and Significance

Ambient artificial intelligence (AI) scribes are increasingly integrated into electronic health record (EHR) systems and have changed documentation workflows in pediatric subspecialty care. 1 2 Early evaluations demonstrate potential reductions in documentation burden, improvements in usability, and variable effects on clinician experience (including reduced burnout). 3 4 5 6 7 8 9 However, large-scale analyses show mixed effects: Liu et al observed no overall reduction in documentation time, 1 while Haberle et al found increased engagement but unchanged patient experience. 10

Pediatric hematology–oncology presents a demanding environment requiring synthesis of complex information, including chemotherapy protocols, supportive care plans, detailed treatment exposures, and anticipatory guidance. 11 12 New patient consultations may encompass months of preceding evaluations, imaging reports, operative summaries, and evolving diagnostic impressions, leading to lengthy, detailed notes. As a result, clinicians frequently complete documentation during “pajama time,” with notes finalized at home after clinical hours. 13 When time is scarce, the level of detail captured may not reflect nuanced experiences shared by patients and families during visits, and subtle concerns may be inadvertently omitted. This environment provides a stringent test of ambient AI documentation tools.

Our division implemented Dragon Ambient Experience (DAX) Copilot (Nuance, Microsoft) to address documentation challenges. Given low overall adoption, this evaluation focuses on providers with repeated use to assess early feasibility, performance, and workflow effect.

## Objectives

This study aimed to evaluate early use of DAX Copilot in a pediatric hematology–oncology division by examining:

1. Patterns of adoption and use.
2. Differences in documentation time between DAX and non-DAX encounters.
3. Effects on documentation timeliness.
4. Implementation considerations relevant to workflow integration and usability.

## Methods

### Quantitative Evaluation of Documentation Time and Timeliness

DAX implementation followed a structured, institution-wide rollout process. Following an ambulatory pilot phase, the tool became broadly available across outpatient settings on January 15, 2025. Standardized training resources included on-demand video tutorials, detailed quick-start guides, webinars, and helpdesks. The pediatric hematology–oncology division included 29 providers (15 attendings, 9 fellows, 5 APPs), all of whom were eligible to use DAX. No pediatric hematology–oncology-specific customization or targeted training was conducted.

We conducted a retrospective cohort evaluation of outpatient encounters documented between January and July 2025. Encounters using DAX were identified through system metadata. For analysis, encounters were categorized as ≤40 or >40 minutes to distinguish established shorter follow-up visits from longer new or complex visits. Because only six providers used DAX more than twice, analyses comparing documentation time and timeliness were limited to this subgroup. Documentation time was measured as active time spent editing notes. Documentation timeliness was assessed as same-day closure and closure within 7 days. Implementation processes were reviewed, and structured user feedback was synthesized to highlight workflow considerations, accuracy issues, and privacy challenges encountered during deployment.

Descriptive statistics were used to summarize adoption patterns, documentation time, and timeliness outcomes. Documentation time exhibited right-skewed distributions and is reported using medians and interquartile ranges (IQRs). For inferential analyses, documentation time was log-transformed and analyzed using mixed-effects linear regression models with provider-level random intercepts to account for clustering of encounters within clinicians. Models included DAX use, appointment length category, their interaction, and appointment hour as fixed effects.

Timeliness outcomes (same-day closure and closure within 7 days) were analyzed using mixed-effects logistic regression models with provider-level random intercepts and adjustment for appointment length and appointment hour. Results are reported as effect estimates with corresponding confidence intervals and *p* -values. All analyses were conducted using Stata (StataCorp, College Station, Texas, United States).

### Qualitative Feedback

To better characterize clinician experience with DAX, brief qualitative feedback was solicited from repeat users via structured email prompts. Providers were asked to comment on workflow integration, perceived strengths and limitations, documentation timeliness, accuracy in complex encounters, trust in higher-stakes conversations, and factors influencing adoption. Six repeat users (100%) provided feedback. Responses were summarized descriptively to identify common themes regarding usability, workflow fit, and perceived accuracy.

## Results

### Adoption Patterns

Across 11,544 outpatient encounters, DAX was used in 427 encounters (3.7%). Ten of 29 providers (34.5%) used DAX at least once. Six providers contributed more than two DAX-assisted encounters during the study period and were classified as repeat users. Among encounters contributed by repeat users ( *n* = 1,615), DAX was used in 25.6% of visits. Adoption intensity varied substantially, with the number of DAX-assisted encounters per provider ranging from 21 to 293.

### Documentation Time

For shorter appointment slots (≤40 minutes), documentation time was substantially lower for DAX encounters than for non-DAX encounters (median = 11 [IQR: 13] vs. 24 [IQR: 30] minutes). Descriptive statistics for documentation time by DAX use and appointment length are presented in Table 1 . In mixed-effects models accounting for clustering by provider, DAX use was associated with approximately 30% lower documentation time for shorter visits (95% CI, 19.9–38.4%; *p* < 0.001). Results of the mixed-effects linear regression model are presented in Table 2 .

**Table 1:** Documentation time and timeliness by Dragon Ambient Experience use and appointment length

	DAX encounters ( *N* = 414)	Non-DAX encounters ( *N* = 1,201)
Appointment length (min)		
≤ 40	215	824
> 40	199	377
Documentation time, min (median [IQR])		
≤ 40-minute appointments	11 [13]	24 [30]
> 40-minute appointments	19 [38]	36 [37]
Documentation timeliness		
Same-day closure	32 (7.7%)	258 (21.5%)
Closed within 7 days	191 (46.1%)	726 (60.5%)

Abbreviations: DAX, Dragon Ambient Experience; IQR, interquartile range.

**Table 2:** Mixed-effects model of documentation time (log minutes)

Variable	Coefficient (β)	95% CI	*p* -Value
DAX (ref: no DAX)	−0.353	−0.484 to −0.222	<0.001
Appointment length: 60–120 min (ref: 15–40)	0.467	0.367 to 0.566	<0.001
DAX × 60–120-min appointment	0.010	−0.177 to 0.197	0.917
Appointment hour	0.006	−0.010 to 0.023	0.459

Abbreviations: CI, confidence interval; DAX, Dragon Ambient Experience.

For longer appointment slots (>40 minutes), the mean documentation time was similar between DAX and non-DAX encounters, and the interaction between DAX use and appointment length was not significant, indicating no consistent benefit for longer encounters. Provider-level heterogeneity was evident, with documentation-time patterns varying across clinicians.

### Timeliness

In unadjusted analyses, same-day note closure occurred in 9.4% of DAX encounters compared with 34.2% of non-DAX encounters, and closure within 7 days occurred in 47.8% versus 80.8%, respectively. However, in mixed-effects logistic regression models accounting for clustering by provider, appointment length, and appointment hour, DAX use was not independently associated with same-day note closure or closure within 7 days. Results of the mixed-effects logistic regression models are presented in Table 3 .

**Table 3:** Mixed-effects logistic regression models of documentation timeliness

Same-day note closure			
Variable	**OR**	**95% CI**	***p*** **-Value**
DAX (ref: no DAX)	0.68	0.35–1.31	0.247
Appointment length: 60–120 min (ref: 15–40)	0.31	0.19–0.48	<0.001
DAX × 60–120-min appointment	1.29	0.46–3.62	0.624
Appointment hour	0.80	0.74–0.87	<0.001
**Note closed within 7 days**			
Variable	**OR**	**95% CI**	***p*** **-Value**
DAX (ref: no DAX)	1.14	0.78–1.66	0.500
Appointment length: 60–120 min (ref: 15–40)	0.37	0.27–0.50	<0.001
DAX × 60–120-minute appointment	1.34	0.77–2.32	0.305
Appointment hour	0.92	0.87–0.96	<0.001

Abbreviations: CI, confidence interval; DAX, Dragon Ambient Experience; OR, odds ratio.

Longer appointment slots were consistently associated with lower odds of timely closure, and documentation timeliness varied by time of day. These findings suggest that downstream clinical workflows, such as visit complexity or pending laboratory results, rather than the use of ambient scribing technology, primarily drive note-closure timing. Overall, DAX did not independently accelerate chart closure in this setting.

### Provider-Reported Strengths and Limitations

Repeat users consistently reported that DAX was most beneficial for new patient encounters, particularly for generating comprehensive history of present illness documentation. Several clinicians noted that DAX-assisted drafting reduced reliance on handwritten notes and after-hours documentation. One provider reported eliminating routine weekend documentation sessions after adopting DAX.

Providers emphasized that DAX was particularly effective at transcribing past medical, social, and family histories and organizing content into appropriate note sections. Some noted that performance appeared to improve with continued use.

Reported limitations were primarily related to workflow integration rather than transcription accuracy. Clinicians described needing to manually populate discrete EHR history fields (medications, allergies) to support shared documentation across providers. In encounters when tangential rapport-building dialogue occurred, DAX occasionally transcribed non-clinical discussions that required editing. One provider noted occasional difficulty distinguishing speakers when the parent and patient were the same gender.

Importantly, repeat users did not report frequent misattribution of clinically significant statements or omission of pediatric hematology–oncology-specific plan elements. Most respondents expressed high confidence in the accuracy of DAX-generated content, including during higher-stakes encounters, while emphasizing the need for routine clinician review and refinement prior to note finalization.

Documentation timeliness was influenced in part by clinical workflow rather than DAX performance. One provider reported intentionally delaying note closure pending complex laboratory results, despite same-day completion of narrative documentation. Other respondents noted that DAX-supported drafting improved recall of visit discussions when notes were finalized later.

## Discussion

The early implementation of DAX in pediatric hematology–oncology demonstrates both promises and sociotechnical constraints of ambient AI scribes when deployed in high-complexity clinical environments. While some repeat users experienced meaningful reductions in active documentation time during shorter encounters, efficiency gains were inconsistent, and overall adoption remained low. Together, these findings highlight a central informatics principle: The effect of AI-enabled documentation is shaped not only by model performance but also by workflow alignment, user trust, and integration with existing EHR tools. In this context, reductions in documentation time were observed primarily for shorter visits, whereas documentation timeliness was not meaningfully affected, highlighting the role of downstream clinical workflows in shaping observed outcomes.

Clinicians perceived notable communication benefits when using DAX. Ambient transcription reduced the need for manual note-taking and supported more uninterrupted engagement with patients and families. Prior literature has suggested that ambient AI may enhance perceived connectedness without necessarily improving formal patient-experience metrics; our findings were consistent with this pattern. 7 These observations underscore the potential for ambient AI technologies to shift the human–AI partnership during clinical encounters by allowing clinicians to focus more fully on relational aspects of care.

Although prior literature has described challenges with multispeaker attribution and omission of clinically relevant details in ambient AI systems, repeat users in our cohort reported high confidence in transcription accuracy. 14 Reported limitations were more commonly related to workflow integration and editing requirements than to clinically significant documentation errors. In particular, clinicians described the need to manually populate discrete EHR fields and remove tangential conversational content, rather than correct substantive medical inaccuracies. These findings suggest that, in this setting, workflow alignment and structured data integration were more salient barriers to adoption than transcription fidelity.

These findings reinforce the need for deeper integration between ambient AI systems and the EHR's underlying data structures. DAX does not retain Epic (Verona, Wisconsin, United States) “dot phrases,” including SmartTexts and SmartPhrases, which are user-defined templates and text macros used to rapidly populate structured sections of a note. As a result, clinicians had to manually rebuild their usual templates in each DAX-generated draft, reducing overall efficiency and increasing the cognitive burden of rewriting and reformatting AI-generated text to meet documentation standards. From a sociotechnical perspective, these limitations decreased the perceived “net value” of adopting DAX, contributing to low sustained uptake. The experience of a small group of high-volume users suggests that proficiency and workflow adaptation can support more consistent use, but broader adoption will require improved alignment between system output and clinical expectations.

Adoption patterns appeared influenced by individual comfort with new technology and perceived workflow benefit. Early adopters reported strong satisfaction and actively promoted DAX use among colleagues and trainees, suggesting that peer modeling and local champions may play an important role in diffusion within specialty settings. These findings align with established technology adoption frameworks in health care, such as the Unified Theory of Acceptance and Use of Technology (UTAUT), in which perceived usefulness and social influence drive sustained use. 15

Privacy and trust also shaped clinician use. Pediatric hematology–oncology encounters often involve sensitive genetic, prognostic, or adolescent confidentiality topics. Although DAX complies with HIPAA requirements, clinicians must be cautious when reviewing and finalizing notes containing sensitive information. This reflects a core challenge in human–AI teaming: Users must trust not only the accuracy of generated content but also its appropriateness for inclusion in the medical record.

Taken together, these findings highlight key design and implementation lessons for ambient AI scribes. Technologies intended for complex care environments must support both narrative fidelity and structured-data accuracy, incorporate safeguards for multispeaker attribution, and integrate seamlessly with specialty-specific EHR workflows. Beyond model-level improvements, the implementation strategy must address training, expectation management, and workflow redesign to support the human–AI partnership inherent in ambient documentation.

Future development should focus on three informatics priorities: (1) Enhancing structured plan-generation capabilities through tighter integration with clinical data models and oncology protocols; (2) improving integration with EHR data structures and discrete documentation fields; and (3) optimizing workflow fit through better alignment with specialty-specific documentation practices. Future work could examine whether training ambient AI systems on individual providers' prior EHR notes improves acceptability by better aligning generated documentation with personal writing style. Although this additional tuning may introduce additional cost, understanding this tradeoff could be important in increasing provider acceptance of the generated output. Collaboration among developers, informaticians, and pediatric subspecialists will be essential to ensure that ambient AI scribes evolve in ways that support both efficiency and clinical integrity.

## Conclusion

DAX showed potential to reduce active documentation time in shorter encounters, but adoption was limited, and benefits were inconsistent. Workflow integration challenges, limited support for structured oncology workflows, and the need for careful review constrained its usefulness. These findings highlight that ambient AI scribes must integrate more tightly with specialty-specific documentation needs and existing EHR tools. Ongoing collaboration among clinicians, informaticians, and developers will be essential to improve workflow fit and realize the full potential of ambient AI documentation systems.

## Notes

**Clinical Relevance Statement**
Ambient AI scribes may reduce documentation time for certain encounter types, but require strong workflow alignment to yield consistent benefit. Early implementation findings underscore the need for specialty-specific customization and careful evaluation of accuracy and usability. These insights can guide informatics teams deploying AI-assisted documentation tools in similarly complex clinical environments

## Notes

**Multiple-Choice Questions**

1. Which factor most strongly supports the successful adoption of ambient AI scribes in clinical practice?
  - Increasing the number of available clinic rooms.
  - Ensuring alignment between AI-generated notes and clinician workflows.
  - Standardizing clinic schedules to 20-minute visits.
  - Reducing the number of EHR templates available.
  **Correct Answer** : The correct answer is option b. Ambient AI scribes perform best when their outputs integrate smoothly with the documentation tools clinicians already rely on. Workflow alignment allows clinicians to maintain efficiency, trust the tool, and avoid additional steps such as reformatting or rebuilding templates. Physical space, scheduling patterns, or fewer templates do not address the core sociotechnical need for integration between AI and real-world clinical work.
2. Which approach best supports clinician comfort when adopting an AI documentation tool?
  - Allowing clinicians to explore the tool at their own pace with optional guidance.
  - Replacing all documentation workflows immediately with AI-generated notes.
  - Removing access to traditional documentation tools.
  - Allowing only trainees to use the AI tool initially.
  **Correct Answer** : The correct answer is option a. Clinicians adapt more easily when they can experiment gradually, maintain autonomy, and access help as needed. Abrupt replacement of workflows or restricting access undermines trust and increases resistance.
